# Foreign Summary

**Published:** 1839-11-23

**Authors:** 


### FOREIGN SUMMARY.

### Velpeau's Clinical Lectures on Ophthalmia. No. X.

*Sequelæ and Complications of Keratitis.*

*Softening of the Cornea.*—Whenever a tissue becomes inflamed, the cohesion that exists between the elements of which it is formed, is always more or less diminished, according to the nature of the tissue. It is not, however, to this phenomenon, which is too general and too well known to require comment, but to a peculiar form of softening of the cornea—a form which has not yet been sufficiently studied by authors, that I wish to draw your attention.

After inflammation of the cornea, the tissue of that organ softens in some instances to such an extent that its form may be entirely altered. Thus, M. Mirault mentions a case in which the pressure of the eyelids caused the cornea to lose its convex form, and to become perfectly flat. It may be pressed forwards, and become elongated, so as to hang betweeen the eyelids, as in a case of M. Stæbers. It may also project, and assume a conical figure, when the softening occupies the centre, or forms a half transparent yellowish brown tumour. When the softening is only partial, and exists in different parts of the membrane, you will sometimes see on its surface several tumours, which appear, by their brownish colour, as if formed by the iris. Some cases have even been observed in which the softening of the cornea was so great, that its form changed under the influence of muscular contraction.

I have occasionally met with a species of softening of the cornea, which is but imperfectly known; indeed, I do not know whether it has ever been properly described. The cornea, the tissue of which has become extremely rarefied, forms between the free margins of the eyelids, a black, brown, or reddish tumour, projecting like a large staphyloma of the iris. This tumour is soft, insensible and radiated. I first observed it in two women, nurses at the Hôpital de la Maternité, who were both affected with a chronic vaginal discharge. They were both of a lymphatic constitution, deteriorated by poverty and bad living, and had been attacked without any appreciable cause, with violent purulent ophthalmia. The flaccidity of the muscles, the copper-coloured appearance of the face, and the vaginal affection, led me to suppose that they were affected with syphilis, although they positively denied this to be the case.

These various forms of softening of the cornea are nearly always attended with serious consequences to the functions of the eye. The cornea being generally more or less deformed, even in the most favourable cases, vision is necessarily disordered.

When the disease does not depend on a specific cause, astringent collyria constitute the best medication. If, on the contrary, the affection appears to be of a specific nature, the treatment must be modified accordingly. Thus, in the cases I have just mentioned, emollient and astringent applications having failed, I had recourse to antisyphilitics. This treatment very soon arrested the progress of the disease, and by then cauterizing with the nitrate of silver, the cure was completed.

*Gangrene* of the cornea, considered merely as the consequence of inflammation of that membrane, is of extremely rare occurrence, so much so, indeed, that I have never yet met with a case. Saunders, it is true, says that he has often seen keratitis followed by gangrene; but the cases he brings forward, as also the facts mentioned by Mr. Lawrence, appear to me to apply more to suppuration and softening of the cornea than to gangrene of that organ. Beclard and M. Mirault in France have, however, each published a case in which the gangrene certainly appears to have been the consequence of inflammation. If we are to look upon all these cases as being really instances of gangrene, we may conclude, that, existing as a termination of keratitis, gangrene does not always give rise to the perforation of the cornea, as the superficial lamellæ only may be disorganized. An albugo, or leucoma, is however, the necessary consequence of such a lesion.

After keratitis, *vegetations* are occasionally seen on the cornea. The situation, and aspect of these vegetations are exceedingly variable; sometimes they occupy the circumference of the cornea; sometimes they are only met with on the centre of that membrane. When they are situated near the circumference of the organ, they may either entirely surround it, or merely show themselves on a segment of its margin. They advance more or less on the cornea, are of a grayish or reddish colour, of variable size, and have the flattened, granular appearance of the papillæ of the tongue. When they appear in the centre, their size is smaller and their colour less vivid. They may either be separate or clustered together.

The only rational mode of treatment that can be resorted to consists in excision and cauterization. If the vegetations are small, cauterization with the nitrate of silver is generally all that is required; if they are large and hard, they should be excised with a lancet or a cataract needle, and the exposed surface then cauterized.

Under the name of aphthoïdal papulæ I have sometimes described to you a species of vegetation which I have often observed, and which I think constitutes the *poros* or *porosis* of surgical writers. These papulæ, which are generally found at the union of the sclerotica with the cornea, occupying the extremities of the transverse diameter of the eye, have the appearance of a variolous pustule. M. Sichel, who looks upon them as indicating scrofulous ophthalmia, says, that they are never seen beyond the limits of the sclerotica. This assertion, however, is not correct, for I have seen them on the surface of the cornea, as far as two lines from its circumference. When they appear near the edge of the cornea they are hard, adherent, of variable size, of a pale red colour, and seem formed by the conjunctiva and the cellular layers immediately underneath. The summit of the papula soon becomes flattened, depressed, and assumes a yellowish tinge, having exactly the same appearance as the aphthæ of the mouth. Those which are met with on the cornea are generally narrow, and form the summit of a vascular triangle or pyramid. These papulæ have been described by some authors as ulcers, although, in reality, they are but simple aphthæ; the excavation which they present not depending on loss of substance, but on thickening of their parietes. Mr. Wardrop says that they are most frequently met with in winter, and in cold wet weather; I have myself seen them in every season of the year.

The presence of these papulæ, which generally disappear with the inflammation which has given rise to them, is not attended with any danger. The astringent collyria, dry or liquid, nearly always prove sufficient to effect a cure; but cauterization with the nitrate of silver is decidedly the most efficacious remedy.

*Abscesses.*—When the cornea is inflamed, of a white, semiliquid, puriform matter, eff occasionally takes place between the lamellæ of that membrane, as most of you, no doubt, have frequently had an opportunity of observing. There has been much discussion to ascertain whether this matter, which Scarpa calls *concrescible lymph*, is of a purulent nature or not; or whether, indeed, the cornea is susceptible of suppuration. In my opinion the dispute is merely one of words. Each tissue may be said to have its own mode of suppurating. The suppuration of a mucous or of a serous membrane does not, as you are well aware, furnish a pus exactly similar to that which is produced by the suppuration of cellular tissue or of the skin. Is it not, therefore, perfectly rational to allow that suppuration of the cornea may present peculiar characters—characters which are not met with in the suppuration of other tissues? In short, it is of but little importance whether the matter be called pus or coagulable lymph, provided the characters which it presents, when effused between the lamella of the cornea, be carefully noted.

These abscesses nearly always exist as sequelæ of interstitial keratitis. Their characters differ, according as they are situated near the sclerotica, or in other parts of the cornea. When they do not occupy the circumference of the organ, their form, size, and position, may vary considerably. Sometimes they are globular, sometimes they are flattened; their size is generally about that of a millet seed: in some instances, however, they appear under the form of yellowish patches, of variable size, surrounded by an opaline areola. They may occur in any part of the cornea, but are more frequently met with in its lateral and inferior regions, than above the pupil. If the abscess is superficial it soon opens externally; if, on the contrary, it is deep-seated, several weeks may elapse before such an event takes place; nor does it always thus terminate. The posterior lamellæ of the cornea may be perforated, and the abscess empty itself into the anterior chamber; this is, however, seldom the case. It is generally considered advisable not to open these abscesses, but to allow them to open spontaneously. Such is certainly the most rational plan, but I am inclined to think, from several experiments which I have made, that the incision of an abscess of the cornea is scarcely ever attended with serious consequences; that it is not dangerous, as has been asserted, but merely useless. That the operation should be useless is easily accounted for, when we consider that the matter which forms the abscess, being half concrete, and extremely adherent to the tissue of the cornea, does not escape through the opening which has been made; but, when the abscess is of a certain size—when it is situated opposite the pupil—and does not seem likely to open alone, it is necessary to use the lancet, as its desiccation might give rise to a permanent opacity of the cornea.

When abscesses of the cornea appear near the sclerotica, they assume a semi-lunar shape, and have received the name of onyx, from the resemblance which has been found between them and the lunula which is seen at the attached extremity of the nails. Some authors have asserted that they are only met with on the inferior segment of the cornea, but this is not correct, as I have seen them on every portion of its circumference. These abscesses frequently terminate by resolution, but sometimes they extend, on the contrary, in such a manner as to give rise to purulent infiltration of the entire organ. They may open externally, in which case they are followed by a deep incised ulcer, or internally into the anterior chamber, thus giving rise to hypopium. The treatment of this class of abscesses is the same as that of the preceding, with the exception that the use of the lancet seems really to retard the cure. Every thing must be done that is calculated to promote the resolution of the effused matter. The remedies which are likely to produce this effect are those which are employed in the treatment of acute keratitis.

Ulcers of the cornea constitute an important complication of keratitis, and have ever attracted great attention from ophthalmologists. They are generally the consequence, but may also be the cause of acute inflammation of the cornea, and as a thorough knowledge of their various modifications may throw some light on the treatment I intend to study them with care. On examining attentively the various ulcers which are observed, and on taking into consideration their seat, and their mode of development we may, I think, establish five species, the characters of which are sufficiently striking to distinguish them easily from one another. In the last century, many more were described by surgical writers, but those which I am about to enumerate comprise all the forms of ulceration which are really met with in practice. They are as follows:—

The nephelion.
The lymphatic or plastic ulcer.
The bothrion.
The epicauma or burning ulcer.
The incised ulcer (*en coup d'ongle.*)

Were the depression occupying the summit of the papulæ, which I described when speaking of vegetations of the cornea, to be considered as an ulcer, we should have to admit a sixth species; but you are well aware that the papulæ in question are merely aphthæ, the product of diphtherical inflammation.

The *nephelion* is an extremely superficial ulcer, and generally appears on the centre of the cornea. It is seldom single, several ulcers of the same species being nearly always clustered together. There is also nearly always a slight effusion of lymph between the lamellæ of the cornea around the ulcer, which renders it extremely difficult to distinguish it from a nebula. If, however, you examine the cornea attentively with a lens, or even with the naked eye, you will find in the centre of the slight opacity, one or more small excavations in the form of a cupola. This kind of ulcer, called by the ancients *achlys* or *caligo*, is principally met with in children and young people. It is rare in persons above thirty, and is never seen in those who are above forty.

When an abscess or a collection of coagulable lymph formed between the lamellæ of the cornea, opens externally, the small wound to which it constitutes what has been called the plastic ulcer. This species of ulcer is more frequently met with in practice than any other. The size varies from that of a pin's head to that of a millet-seed. The edges are jagged, irregular, festooned, and the bottom is uneven. It is slow in cleansing, and the tissue of the cornea being consequently protected from the contact of the air, there is less photophobia or epiphora than in the other forms of ulceration. It is worthy of remark, that the opacity to which the plastic ulcer gives rise, is generally more extensive and deeper seated than that which follows other ulcers. When it is situated near the circumference of the cornea, it generally rests on the summit of a vascular patch of a triangular or pyramidal form, the basis of which is on the conjunctiva. The vascular patch, which at first might be taken for pterygium, is sometimes thick and moveable; sometimes, on the contrary, it is thin, or seems to belong entirely to the cornea. The plastic ulcer is considered to be one of the characters of scrofulous ophthalmia. It is more especially observed, it is true, among young people and persons of a scrofulous constitution, but this is by no means a general rule. The numerous cases of the plastic ulcer which we have had lately, or have yet, in our wards, must have proved to you that it may occur in all ages and in all constitutions.

The *Bothrion* ulcer commences by a phlyctena, which, being nearly transparent, often passes unperceived. In the course of a day or two this phlyctena breaks, and is succeeded by an excavation. The ulcer which is thus formed may be either superficial or deep-seated. When it is superficial, it is generally of a circular shape; when it is deep-seated, it often assumes the form of a tear, with the point turned outwards. The bottom is transparent, and differs so little from the tissue of the cornea, that when the ulcer is superficial, it is often only by looking at the eye sideways that you can discover its presence. The small vessels which are distributed to the neighbourhood of the ulcerated surface, arise from the deep-seated vascular layer. These vessels are often larger and more numerous around the ulcer than on the sclerotica, and sometimes appear all to arise from a common trunk, which is plainly seen on the margin of the ulcer. The dread of light, and the shedding of tears, are carried to a greater extent in this form of ulceration than in any other.

The progress of these ulcers is rather singular; the vascular injection of the cornea and other tissues of the eye gradually diminishes, and at last disappears, as likewise the photophobia and epiphora. The ulcer, however, still retains the same excavated appearance, and often remains in this state for a considerable length of time; the slight cavity it forms may even become indelible, in which case the patient is continually exposed to a renewal of the inflammation.

In this, and in the preceding form of ulceration, the tissue of the cornea is sometimes entirely destroyed. When this is the case you will see a small bulla arise gradually from the bottom of the ulcer, until it slightly protrudes on the free surface of the cornea. The small tumour or myocephalon, which is thus formed, is owing to the hernia of the membrane of the aqueous humour, and must not be confounded with hernia of the iris, or the true myocephalon.

The *Epicauma* or serpiginous ulcer, thus denominated by the ancients, and described by Ware under the name of abrasion of the cornea, at first certainly bears more resemblance to an excoriation of the superficial lamellæ of that membrane than to a real ulcer. It is often met with in acute keratitis, when it appears under the form of a slight abrasion of the cornea, sometimes several lines in width, which is generally situated near the circumference of the organ, and which gradually advances towards the centre, without, however, ceasing to be superficial. In some instances, I have known it to occupy the entire circumference of the cornea, the central portion only remaining intact. Were I to be guided by my own experience, I should say that the superior half of the membrane is more especially the seat of this kind of ulcer. If the epicauma in this, its first period, be left to itself, it gradually sinks deeper into the tissue of the cornea, and at last assumes the appearance of a real ulcer. When there are several of these ulcers present at the same time, the aspect of the cornea is peculiar; it appears as if it were covered with facets. There is little or no vascularity of the subjacent tissue of the cornea, or of that which surrounds the ulcerated surface; the conjunctiva, on the contrary, is, generally speaking, considerably vascularized. There is, nearly always, considerable photophobia and epiphora; but the intensity of these symptoms varies with the depth and extent of the lesion. The pain, which is often severe, is superficial, and does not irradiate to the orbit. When the ulcer is healed, there remains a slight opacity of that part of the cornea on which it was situated, and vision is consequently more or less disordered; but this opacity diminishes with time, and sometimes disappears entirely.

The *incised* ulcer, although not separated from the other species of ulcers by the ancients, is well worthy of a separate description. It has been described with care by Mr. Lawrence, in his work on Venereal Diseases of the Eye, as a symptom of venereal ophthalmia; I have, however, often met with it, when I could not possibly attribute it to a venereal affection. This ulcer is nearly always found at the circumference of the cornea, near the sclerotica, in the same situation as the senile zone, under the form of an arc of a circle, from one to four lines in length, but scarcely ever more than a line in width. It has two lips or edges, which do not offer the same characters. The outer or sclerotic lip appears as if it had been cut perpendicularly, and is red and vascular, owing to its being formed in a great measure by the conjunctiva, which is thickened and injected. The inner lip, on the contrary, is slightly bevelled, and remains at first perfectly transparent, unless there be suffusion of that portion of the cornea. In a short time, however, vascular filaments appear in the tissue of the cornea underneath and around the ulcer; you will even sometimes see vessels perfectly isolated in the ulcerated cavity. This kind of ulcer never advances towards the centre of the membrane; its depth, however, gradually increases, and the arc which it forms extends progresively along its circumference. When there are several, they may unite so as to circumscribe and isolate the cornea entirely. The incised ulcer is always accompanied by photophobia and epiphora; these symptoms are, indeed, often very intense. It gives rise to vegetations of the cornea more frequently than any of the other species of ulcer.

The various species of ulcer, which we have thus briefly examined, are nearly always the result of keratitis. They may, however, in some instances, be the cause of the inflammation of the cornea; as, for instance, when a metallic particle flying into the eye, becomes implanted into the cornea, and, on falling, leaves behind it a bothrion ulcer.

The characters which I have given you, if borne in mind, will always enable you to distinguish these ulcers from one another—a point of great practical importance in the treatment of ulcerated keratitis, for you must not suppose that the distinctions I have established are merely theoretical.

---

*Narcotine as a substitute for Quinine.*—The muriate of narcotine is recommended in the Calcutta Medical Journal as a substitute for quinine. A strong mass of testimony will be required to induce the prudent practitioner to experiment with any new remedy, to the exclusion of, perhaps, the most valuable and certain article in the materia medica."

---

*Nitrate of Silver in Phlogosis of the Mucous Membranes.*—M. Boudin has extended the application of nitrate of silver to the cure of inflammations and ulcerations of the ileum, which constitute one of the most constant lesions in typhoid fevers. When diarrhœa is the principal symptom, he administers the nitrate in enema, in the dose of from two to eight grains dissolved in six ounces of distilled water; and when gastric symptoms predominate, he gives it by the mouth, in pills, in the dose of a fourth to half a grain; and when the whole gastro-intestinal mucous membrane appears phlogosed, he combines the two modes of administration.—*Edinburgh Med. and Surg. Journ.*, *from Journal des Connaissances Medicales Pratiques*, May, 1838.

---

*On the Disorders of the Brain connected with Diseased Kidneys.* By Thomas Addison, M. D. (Guy's Hospital Reports, No. vi, April, 1839.)—According to Dr. Addison's experience, cerebral affections, connected with renal disease are marked by a pale face, a quiet pulse, a contracted or undulated and obedient pupil, and the absence of paralysis.

He distinguishes five different forms of cerebral disorder. These are:

1. A more or less sudden attack of quiet stupor, which may be temporary and repeated; or permanent, ending in death. This form of cerebral disorder appears to be characterized by a mere torpidity of manner, sluggishness of intellect, and tendency to quiet stupor, and is met with of all degrees of severity up to its most exquisite form, when there is complete insensibility to all surrounding objects. There is, however, no paralysis, no laborious or stertorous breathing, and no convulsions, the face is pale, and the pulse quiet. This appears to be the least formidable variety of cerebral disorder attending renal disease, and is chiefly connected with temporary derangements of the functions of the kidneys. Thus it is not unfrequently observed at an early period in the progress of scarlatina, and in cases of fever, as also in retention of the urine from stricture or calculus. In such instances the symptoms pass away and the patient recovers.

2. A sudden attack of a peculiar modification of coma and stertor, which may be temporary, or end in death. The first form of cerebral affection sometimes passes into this, which is the serous apoplexy of authors. The coma is, for the most part, complete, so that the patient cannot be roused to intelligence for a single moment. The stertor is peculiar, and in a measure characteristic of this form of cerebral disorder. It has not the harsh, deep, gutteral sound of ordinary apoplexy, but has more of a hissing sound, as if produced by the air striking against the hard palate or the lips, rather than against the velum and throat. The act of respiration is in general more hurried than in ordinary apoplexy. The face is pale, often remarkably so; and this, combined with the peculiar hissing stertorous breathing, has often enabled Dr. Addison to pronounce the comatose symptoms to be dependent on renal disease, without asking a single question.

3. A sudden attack of convulsions, which may be temporary, or terminate in death. The face is pale, though occasionally it is flushed at intervals. The pulse is singularly quiet, but during the convulsions becomes rapid, irregular, and jerking. This form frequently passes into the next variety, which, in fact, is—

4. A combination of the two latter; consisting of a sudden attack of coma and stertor, accompanied by constant or intermitting convulsions. These two last forms of cerebral disorder indicate a more serious affection of the kidneys than the first variety. Dr. Addison has observed them most frequently in cases of renal dropsy after scarlet fever, and in that form of dropsy supposed to arise from direct exposure to cold and damp, and known by the name of inflammatory dropsy. Still, patients often completely and permanently recover.

5. A state of dullness of intellect, sluggishness of manner, and drowsiness, often preceded by giddiness, dimness of vision, and pain in the head, proceeding either to coma alone, or to coma accompanied by convulsions. This form makes its approach in a more gradual and insidious manner than any of the other varieties of cerebral disorder which are connected with diseased kidneys. It is the most stubborn and intractable, as well as most fatal form, and is usually associated with that particular disorganization of the kidney described by Dr. Bright.

*Edin. Med. and Surg. Journ.*

